# The Nasal Solitary Chemosensory Cell Signaling Pathway Triggers Mouse Avoidance Behavior to Inhaled Nebulized Irritants

**DOI:** 10.1523/ENEURO.0245-22.2023

**Published:** 2023-04-10

**Authors:** Ranhui Xi, Sean McLaughlin, Ernesto Salcedo, Marco Tizzano

**Affiliations:** 1 Department of Basic and Translational Sciences, School of Dental Medicine, University of Pennsylvania, Philadelphia, PA 19104-6030; 2 Brain Institute, Florida Atlantic University, Jupiter, FL 33458-2932; 3 Department of Cell and Developmental Biology, School of Medicine, University of Colorado Anschutz Medical Campus, Aurora, CO 80045-2609

**Keywords:** avoidance behavior, bitter, chemical senses, irritant, respiratory, solitary chemosensory cells

## Abstract

The nasal epithelium houses a population of solitary chemosensory cells (SCCs). SCCs express bitter taste receptors and taste transduction signaling components and are innervated by peptidergic trigeminal polymodal nociceptive nerve fibers. Thus, nasal SCCs respond to bitter compounds, including bacterial metabolites, and these reactions evoke protective respiratory reflexes and innate immune and inflammatory responses. We tested whether SCCs are implicated in aversive behavior to specific inhaled nebulized irritants using a custom-built dual-chamber forced-choice device. The behavior of mice was recorded and analyzed for the time spent in each chamber. Wild-type (WT) mice exhibited an aversion to 10 mm denatonium benzoate (Den) or cycloheximide and spent more time in the control (saline) chamber. The SCC-pathway knock-out (KO) mice did not exhibit such an aversion response. The bitter avoidance behavior of WT mice was positively correlated with the concentration increase of Den and the number of exposures. Bitter-ageusic P2X2/3 double KO mice similarly showed an avoidance response to nebulized Den, excluding the taste system’s involvement and pointing to an SCC-mediated major contributor to the aversive response. Interestingly, SCC-pathway KO mice showed an attraction to higher Den concentrations; however, chemical ablation of the olfactory epithelium eliminated this attraction attributed to the smell of Den. These results demonstrate that activation of SCCs leads to a rapid aversive response to certain classes of irritants with olfaction, but not gustation, contributing to the avoidance behavior during subsequent irritant exposures. This SCC-mediated avoidance behavior represents an important defense mechanism against the inhalation of noxious chemicals.

## Significance Statement

With every breath, harmful compounds can assault the nasal cavity, which can cause insults ranging from simple nasal congestion and inflammation to permanent damage or even death. Research has shown that the trigeminal chemosensory system in the nose and nasal solitary chemosensory cells (SCCs) help detect harmful inhaled compounds, evoking physical and chemical protective responses such as respiratory-protective reflexes and inflammatory and immune responses. The SCC-mediated avoidance behavior, reported in detail here, represents an important defense mechanism against inhaling noxious chemicals to minimize mucosal damage. Altogether, the SCC trigeminal and sensory systems, assisted by the olfactory system, represent powerful protective layers against inhalation of harmful chemicals and respiratory damage.

## Introduction

This study aimed to test whether solitary chemosensory cells (SCCs) are implicated in aversive behavior to specific inhaled nebulized irritants and to determine whether SCC-mediated avoidance behavior represents an important defense mechanism against the inhalation of noxious chemicals. Avoidance responses to environments polluted with airborne noxious compounds are innate protective mechanisms that comprise several protective reflexes triggered to expel or avoid the irritating compounds. Typical involuntary protective reflexes include sneeze, apnea, laryngospasm, cough, expiration reflex, and swallowing (Nishino, 2000). Associated with protective reflexes are a series of behavioral responses that help recognize and avoid contaminated environments, preventing inhalation of toxic/irritating agents and reducing any further exposure. Animals can detect various olfactory cues that may be crucial to their survival, including predators and pungent odors. Pungent odors are typically selective trigeminal stimuli. Animals show avoidance behaviors to these odors because of their potential toxicity and exhibit an innate escape response to preserve their life (Blanchard et al., 2001; Capone et al., 2005). Most odorants are able to simultaneously stimulate olfactory and trigeminal systems in the nasal cavity, at least at high concentrations (Brand, 2006). The trigeminal system has long been considered essential in many species as a defense system against potentially toxic or noxious molecules. The trigeminal chemosensory system consists of polymodal nociceptive neurons expressing several different types of ligand-gated receptors (Alimohammadi and Silver, 2000; Ichikawa and Sugimoto, 2002; Dinh et al., 2003; Spehr et al., 2004). These receptors detect irritants that permeate the epithelium, including air pollutants (e.g., sulfur dioxide), ammonia, ethanol, acetic acid, carbon dioxide, menthol, and capsaicin (Purves et al., 2001). Stimulation of the trigeminal chemosensory system evokes respiratory protective reflexes to protect the airways from further exposure (Alarie, 1966; Lundberg et al., 1984; Meggs, 1993; Bryant, 2000; Geppetti et al., 2006). A more recently discovered sensory system in the nose comprises epithelial sentinel cells referred to as solitary chemosensory cells (SCCs), which detect other classes of irritants including bacterial quorum-sensing molecules (e.g., N-acyl homoserine lactones), bitter-tasting compounds (e.g., denatonium, cycloheximide), and odorous irritants (e.g., lilial, citral) that are impermeable to epithelial tight junctions, and therefore unable to reach the trigeminal nerve fibers (Gulbransen et al., 2008; Lin et al., 2008b; Tizzano et al., 2010; Saunders et al., 2014). SCCs are specialized chemosensitive epithelial cells that act as sentinels of the nasal mucosa and use the bitter taste receptor transduction cascade to detect inhaled chemicals (Kaske et al., 2007; Tizzano et al., 2010). Trigeminal nerve fibers innervating the SCCs (Finger et al., 2003) relay SCC responses to the central nervous system and trigger the release of neuropeptides into the nasal mucosa, evoking protective reflexes and pro-inflammatory responses similar to trigeminal activation (Tizzano et al., 2010; Saunders et al., 2014). Nasal SCCs are part of the airway protective response systems. While the SCCs represent an effective layer of protection for the respiratory and deeper airway epitheliums, microvillous cells (MCs) specifically located in the main olfactory epithelium (MOE) are another epithelial cell expressing and using the taste signaling transduction pathway (Lin et al., 2008a; Genovese and Tizzano, 2018). MCs are cholinergic and has been implicated in the chemical responsive to various xenobiotics including bacterial lysate and odorants at high concentrations (Ogura et al., 2011). Lemons et al., describes how in mice lacking the POU homeobox transcription factor Skn-1a (Skn-1a-KO) that eliminates chemosensory cells, including SCCs, MCs, taste receptor cells, and more, a two-week exposure to high-concentration of odor chemicals and chitin powder, there is a significant reduction in their odor and pheromone-evoked odor-evoked electro-olfactogram responses and significant impairment in the olfactory-guided behaviors (Lemons et al., 2017). Thus, MCs contribute to maintaining the olfactory function of the MOE.

Published behavior data focuses primarily on irritants that activate the trigeminal chemosensory system (ammonia, ozone, formaldehyde, etc.; Tepper and Wood, 1985; Wood and Coleman, 1995; Bryant, 2000; Morris et al., 2003; Saunders, et al., 2013; Yonemitsu et al., 2013). Current research on irritants that selectively activate the SCC signaling pathway has focused mostly on protective reflexes, whereas behavior mediated by SCC activation remains poorly investigated. Denatonium benzoate is the bitterest known chemical compound (McLaughlin and Margolskee, 1994) and is used as an aversive agent (bitterant) in dangerous chemicals to prevent ingestion. Denatonium also happens to be a well-known SCC trigger and is considered the prototypical bitter model compound for activating the SCCs T2R pathway (Gulbransen et al., 2008; Tizzano et al., 2010; Saunders et al., 2014).

**Figure 1.**
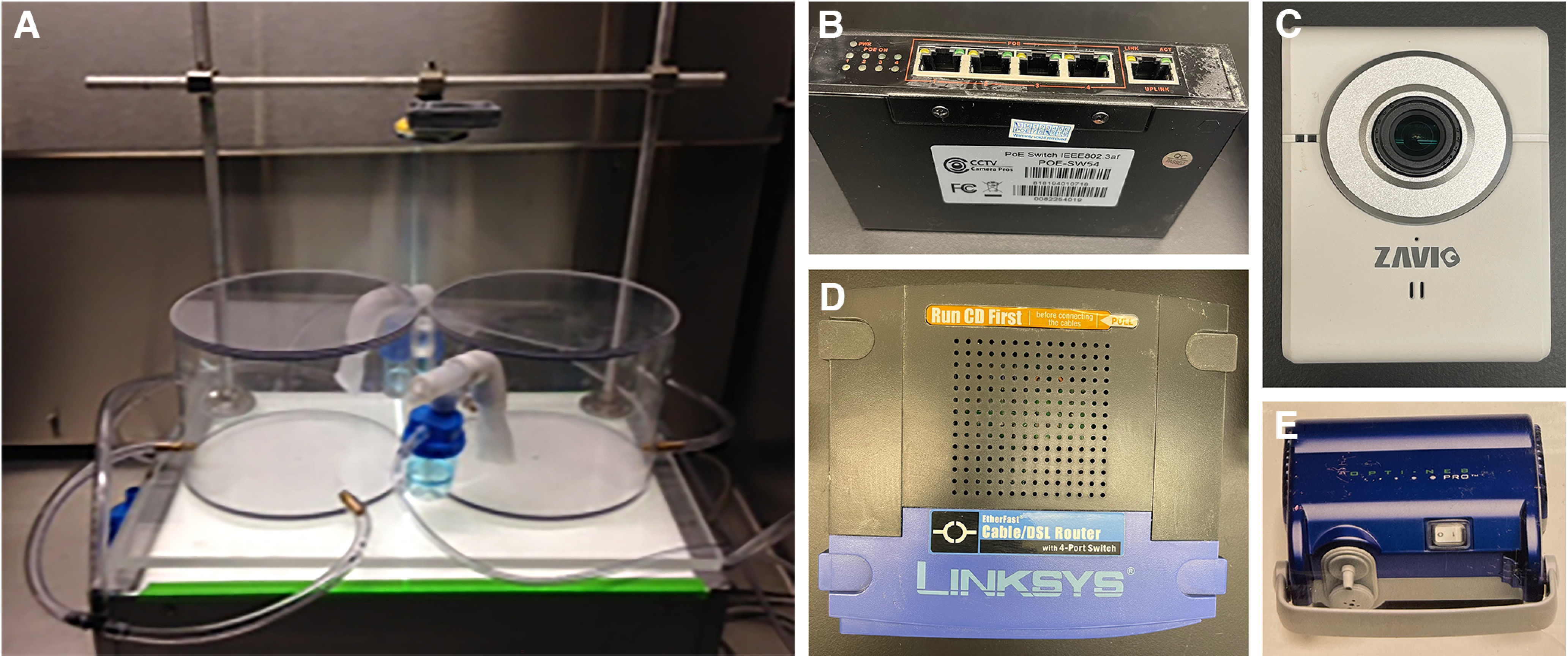
Custom-built dual-chamber forced-choice device used to measure avoidance responses to aerosolized irritants. ***A***, Dual-chamber forced-choice device. ***B***, Camera Power over Ethernet (POE) switch. ***C***, Infrared camera. ***D***, Computer router. ***E***, Nebulizer compressor.

In this study, we tested the role of SCCs in avoidance behavior to aerosolized-nonvolatile irritants using a custom-built dual-chamber forced-choice device (Fig. 1) with two interconnected chambers separated by a small passage to observe mouse avoidance behavior following exposure to harmful compounds in the environment. The hypotheses tested were: (1) SCCs activation by denatonium and other SCC-activators evokes respiratory protective reflexes and avoidance responses; (2) the SCC sensory system is primarily involved in the avoidance responses; and (3) mice improve the avoidance response after repeated exposure to the same SCC-activator, demonstrating learning. The first hypothesis builds on the idea that SCCs can trigger protective reflexes that mice recognize as noxious stimuli, leading to avoidance of the chamber with the nebulized irritant. The second hypothesis implies that the avoidance behavior is mainly mediated by SCCs, with no or little involvement of taste and smell, despite any chemical compound possessing multiple sensory modalities. The third hypothesis implies that the information from SCCs and other sensory systems is integrated to achieve a faster and more efficient avoidance response. To test these hypotheses, we used unique transgenic knock-out (KO) mouse lines that target the SCC pathway, a bitter-ageusic KO mouse, and methimazole to chemically ablate the olfactory sensory epithelium and induce anosmia. In response to dangerous or noxious compounds, the respiratory system triggers a series of airway protective reflexes and evokes behavior responses that ultimately reduce further inhalation of noxious chemicals. Our data indicate that the nasal SCCs are a crucial component in this protective response system.

## Materials and Methods

All animal procedures were performed in accordance with the University of Pennsylvania animal care committee’s regulations and in accordance with the Society’s Policies on the Use of Animals and Humans in Neuroscience Research. Mice of both sexes were used for this study. Mice were provided with group housing and were on a 12/12 h light/dark cycle (zeitgeber time).

### Transgenic mouse models

For this study, we used transgenic knock-out (KO) mice missing elements of the SCC signaling cascade (TrpM5-KO and Gnat3-KO) or missing SCCs entirely (Skn1a-KO), and bitter-ageusic mice that lack bitter, sweet, and umami taste perception (P2X2/3 double KO; Table 1). The genetic deletion of either the G-protein subunit α transducin 3 gene (*gnat3*, also known as α-gustducin; Wong et al., 1996) or the transient receptor potential cation channel subfamily M member 5 gene (*trpm5*; Damak et al., 2006) results in SCC and taste cell function deficits. The Skn1a-KO mouse (Ohmoto et al., 2013) lacks SCCs entirely in the respiratory epithelium because Pou2f3/Skn-1a is a necessary transcription factor for the generation or differentiation of SCCs, as well as type-II taste cells. The purinergic receptors P2X2 and P2X3, which open a cation-selective pore in response to ATP binding, are absent in the bitter-ageusic P2X2/3 double KO mice, making them “blind” to the bitter, sweet, and umami taste. (Cockayne et al., 2005). The TrpM5-GFP mouse was used for the olfactory sensory epithelium ablation experiments to visualize SCCs and microvillous cells.

**Table 1:** Transgenic and knock-out mice used in this study

Abbreviation	Strain^1^	Purpose and function
WT	Wild type	Control
TrpM5-GFP	Green fluorescent protein expression under the promotor for Transient receptor potential cation channel subfamily M member 5 (Trpm5)	SCC, taste cells, and MVC express GFP; immunohistochemical examination of the airway epithelium
Gnat3-KO	G-protein subunit α transducin 3 (gustducin) knock-out	Insufficiency of taste cells and SCCs
Trpm5-KO	Trpm5 knock-out	Insufficiency of taste cells and SCCs
Skn1a-KO	POU (Pit-Oct-Unc) homeodomain transcription factor (Skn-1a/Pou2f3) knock-out	Aplastic Type II taste cells and SCCs
P2X2/X3 Double KO	Purinergic receptors ATP-gated ion channels P2X2 and P2X3 double knock-out	Bitter-ageusic mouse that lacks taste avoidance of bitter substances, or taste preferences for sweet or umami

1 The background strain for each group was C57BL/6.

### Custom-built dual-chamber forced-choice device

To test mice for avoidance behavior, we developed a custom-built dual-chamber forced-choice device that consists of two identical cylindrical chambers connected by a short tunnel (Fig. 1*A*). Each chamber has a compressor and nebulizer (Hudson RCI Opti-Neb Pro 5900) to aerosolize solutions into tiny droplets (Fig. 1*E*). A significant advantage of aerosolized solutions is that the tiny droplets have the same concentration as the original solution. At the opposite side of each chamber, two brass coupler plugs connected to a central vacuum system remove excess aerosol. When developing the dual-chamber device, we calibrated the suction pressure, and the position of nebulizers and vacuum brass coupler plugs to ensure that the suction created by the vacuum maintains separation between the two nebulized solutions within each chamber. The device is completed by a cover for each chamber to create a microenvironment where mice were exposed to a constant flow of aerosolized saline-dissolved compounds. A video camera at the top of the chambers records the movements and behaviors of the mice (Zavio IP F3102; Fig. 1*C*). A Power over Ethernet (POE-SW54; Fig. 1*B*) switch connects the camera to the computer router (Linksys BEFSR41; Fig. 1*D*). At the bottom of the chambers, a transilluminator shielded with several layers of paper creates a lightly illuminated background to facilitate recognition of mouse versus background during video recording analysis using a semi-automated MATLAB script to track mouse movements. During the behavior assay, each chamber was filled with a mist of either saline or an irritant in saline; lights in the room were kept off; and human interference and noise in the room were reduced to a minimum.

### Mouse training and acclimatization

Mice required a training period to acclimate to the chambers, the noise from the nebulizer compressors, and the light from the transilluminator. Mice were placed in the device for 10–20 min/d for 5 d, as explained below. In the first 5 min of each training day, mice were placed in one of the device chambers and were free to explore the two chambers with the nebulizer compressors off and only the transilluminator light on. The compressors were on for the next 5–15 min, and saline was aerosolized in both chambers with no irritant compounds present. Mouse movements and behavior were not recorded during the training sessions. After each training session, mice were returned to their home cage. Mice that failed to move and explore during the first 2 d of training were removed from the experiments, and their training was terminated. Between each training session, the chamber surfaces were flushed with warm water, followed by careful wiping with ethanol wipes and complete drying of the chambers.

### Avoidance behavior assay

Following completion of the training period, one of the saline-filled nebulizers was replaced by an irritant compound in saline, and mice were tested for the avoidance behavior assay to “bitter-tasting” irritant compounds. The irritant compounds used were denatonium benzoate (Den) at 2, 5, and 10 mm concentrations (dose-dependent exposure studies required all three concentrations; in other experiments, only 10 mm was used) or cycloheximide (Cyc) at 10 mm. These compounds and concentrations were chosen from previously published studies (Gulbransen et al., 2008; Tizzano et al., 2010; Saunders et al., 2014). Mice can easily taste Den at 0.1–1 mm concentrations (Finger et al., 2005), while nasal SCCs respond to 10 mm or higher concentrations (Gulbransen et al., 2008; Tizzano et al., 2010; Saunders et al., 2014). Saline (0.9% w/v) was used as a control stimulus and diluent for Den and Cyc. The experimental protocol consisted of three 5 min rounds of exposure in the dual-chamber forced-choice device repeated at three time points (0, 1, and 6 h). At each time point, mice were exposed first to aerosolized saline in both chambers for 5 min to determine the baseline behavioral response (the expected outcome was indifference, spending equal time in both chambers); for the second round of 5 min, by replacing one of the saline-filled nebulizers with one containing an irritant compound solution (expected outcomes either of avoidance, indifference, or preference for the irritant); and for the third 5-min round, by switching sides between saline-filled and irritant-filled nebulizers for the two chambers (to exclude mice preference for a specific chamber). At the first time point (time 0), mice were naive to the irritant compound, and the behavior response was spontaneous. Most chemical compounds activate multiple sensory systems. Den can activate the SCC pathway as well as other sensory systems, such as olfaction and gustation (Chandrashekar et al., 2000; Hallock et al., 2009). Thus, mice can recognize Den through sensory systems other than just SCCs, and build memories of the odor and taste of Den. Therefore, mice exposed to Den should be able to remember and learn from multiple presentations of Den, requiring less time for the avoidance response at subsequent irritant exposures. During each exposure session, mice moved freely between the two chambers. Only one mouse at a time was tested to avoid confounding social behaviors. The location of the irritant-containing nebulizer was randomly selected for the first session. In the following session, the irritant was placed in the other chamber to prevent mice developing a preference for a particular chamber. After each experiment, tested mice were returned to their original cage until the following session. Before the next exposure session, the chamber surfaces were flushed with warm water, followed by careful wiping with ethanol wipes and complete drying of the chambers. Each 5 min exposure session was video recorded, and recordings were analyzed using a MATLAB script to semi-automatically measure the “behavior score” as the ratio between the time spent in the irritant chamber and the total time of the exposure session. The behavior score for the exposure to saline in both chambers generated the baseline behavioral response for each mouse. When in one of the chambers saline was replaced with a stimulus compound, the behavior score at time 0 of the irritant presentation indicates the naive behavioral response. The score for the irritant presentations at time 1 and 6 h represents the behavior response resulting from the SCC-mediated avoidance response added to the possible of other sensory systems, responsible for evoking a faster behavioral response.

### Video tracking mouse movements

**Figure 2.**
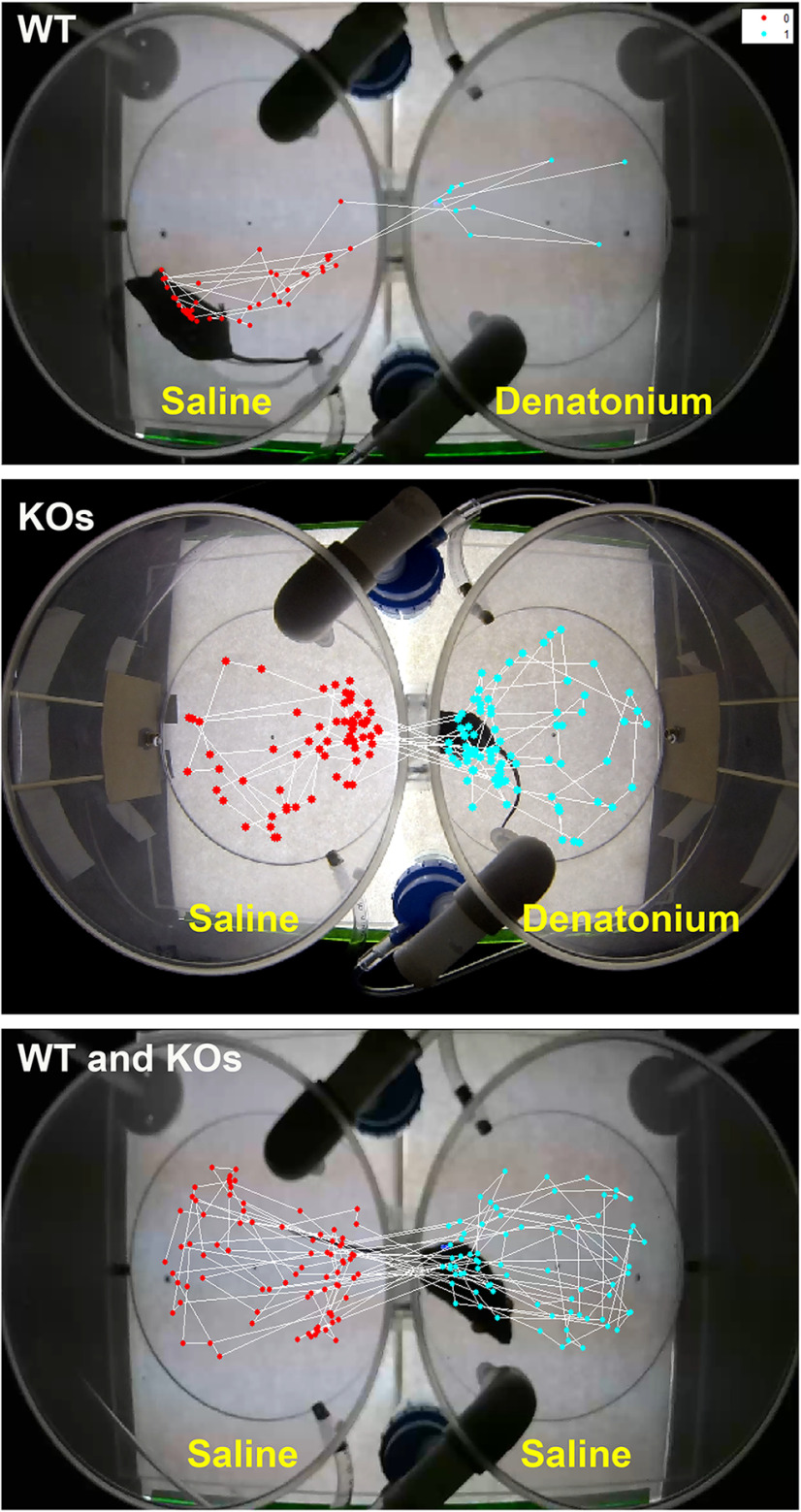
Examples of video recording analysis using the MATLAB script. Top, Wild-type (WT) mice spend the most time in the saline chamber (left), avoiding the denatonium chamber (right). Middle, TrpM5 knockout (KO), Gnat3-KO, and Skn1a-KO mice spend equal time in the irritant and saline aerosolized chambers. Bottom, Both WT and KO mice spend equal time in saline-only aerosolized chambers.

Briefly, a self-contained MATLAB (The MathWorks) app with a user interface was created that allows the user to load a video, define the arena in the video (the area where the mouse can move freely), and then track the movement of the mouse. The user defines the arena by manually tracing the area on one paused video frame. The user then draws a line down the center of the arena to divide it into two separately defined spaces, left and right. Mouse movement is tracked by first designating every 30th frame in the video as a reference frame. Each reference frame is then compared with a comparison frame 15 frames later. Thus, for a 30 frames-per-second (fps) video, one frame for each second of the video is compared with a frame 0.5 s later. Using these frames, we calculated the absolute difference in intensity between the two images. This calculation resulted in a new image where the brightest intensity captured the area with the most change, corresponding to the current location of the moving mouse. This image was then segmented (using the function im2bw) into a binary representation of the mouse. Any segmentation noise in this representation was removed using a morphologic opening operation. Then the centroid of the mouse representation was calculated and saved, along with the current frame location, arena side (left or right), and time stamp. Finally, a plot of the centroids was overlaid onto the arena to visually display the movement of the mouse (Fig. 2). Markers indicating the centroids were plotted along this line were colored differently (cyan vs magenta using gscatter), depending on the arena where they were captured, allowing for visual confirmation of the number of time points collected in the left and right arenas. Time points were compared between the sides to estimate whether the mouse’s movements were random (equal number of time points between sides) or selective to indicate aversion/attraction (more time points on one side vs the other).

### Code accessibility

The MATLAB app described above and example videos are freely available online at https://github.com/salcedoe/T2R_mouse_vid_track. The code was generated using MATLAB 2014a on a Macintosh computer running OS X 10.9 (Mavericks). The code was deployed for data collection on a PC running Windows 7.0. The chamber camera video recordings were captured by a PC running Windows XP.

### Statistical analysis

The “behavior score” value, the ratio of the time in the chamber with the irritant compound solution versus the total time (irritant chamber + saline chamber), was subtracted by 0.5 to create a new zero baseline to simplify the visual representation of either a preference, with value above zero (positive), or avoidance, with a ratio value below zero (negative). For statistical analysis was used GraphPad Prism. One-way or two-way ANOVA was used to compare the differences between multiple groups, supplemented with Tukey’s test for pairwise comparison. Differences were considered statistically significant if *p* < 0.05.

### Methimazole ablation of the olfactory epithelium and microvillous cells

Methimazole (1-methyl-2-mercaptoimidazole, also called thiamazole) is used to treat hyperthyroidism (Astwood et al., 1945) and is known to cause a loss of the sense of smell (olfaction) as a side effect of the treatment (Cooper, 1999). In response to intraperitoneal methimazole injection (MMI), several cell types in the MOE, such as Bowman’s gland cells, sustentacular cells, and olfactory sensory neurons (OSN), display swollen organelles within 4 h, followed by cell detachment from the basal lamina (Bergström et al., 2003). MCs, which, similarly to the SCCs, express elements of the taste transduction signaling pathway and are located superficially in the MOE (Genovese and Tizzano, 2018), are also ablated by MMI during the first few hours postinjection as the superficial MVC cells are detached with the rest of the MOE from the basal lamina. After methimazole injury, the MOE completely regenerates in approximately one month, while OSNs make synaptic contacts with mitral cells in the olfactory bulb at approximately one to two weeks (Suzukawa et al., 2011). We performed ablation of the OE as previously described by intraperitoneal injection of methimazole (75 mg/kg) in 0.9% saline (Håglin et al., 2021). In brief, 75 mg/kg methimazole was prepared under aseptic conditions in sterile physiological saline on the same day as the injections. Methimazole powder dissolves completely without visible precipitate. Mice were injected with the prepared solution at a final concentration of 10 μl/g of body weight. For the behavior experiment with MMI, following the training session, mice were injected with methimazole and 2 d later, mice were tested for avoidance responses as described above. For the experiment of immunohistochemical examination of the airway epithelium, TrpM5-GFP mice were injected with methimazole or saline and euthanized 2 d later. In brief, mice were anesthetized with urethane at a concentration of 1 g/kg of body weight and killed by saline transcardial perfusion followed by perfusion-fixation with 4% paraformaldehyde. The head was dissected, bones decalcified in 10% EDTA for two weeks (replaced with fresh EDTA solution every 2–3 d), and longitudinally cryosectioned (16-μm sections) with tissue sections collected on Superfrost Plus microscope slides. Slides were imaged using an Olympus BX63 microscope.

## Results

A total of 10 mice per condition and experimental group were used in this study (200 in total): 106 (53%) males and 94 (47%) females. Mouse strains used were C57BL/6 wild-type (WT; *n* = 50), TrpM5-KO (50), Gnat3-KO (40), Skn1a-KO (40), P2X2/3 WT (10), and P2X2/3 double KO (10). Experimental groups were as follows: (1) dose-dependent response experiments used C57BL/6 WT (*n* = 30), TrpM5-KO (30), Gnat3-KO (30), and Skn1a-KO (30) mice exposed to denatonium benzoate at 2, 5, and 10 mm concentrations; (2) methimazole ablation experiments used C57BL/6 WT (*n* = 10), TrpM5-KO (10), Gnat3-KO (10), and Skn1a-KO (10) mice exposed to 10 mm denatonium benzoate (Den); (3) cycloheximide (Cyc) experiments used C57BL/6 WT (*n* = 10) and TrpM5-KO (10) mice with 10 mm Cyc; and (4) bitter-ageusic experiments used P2X2/3 WT (*n* = 10) and P2X2/3 double KO (10) mice.

### Nasal solitary chemosensory cells mediate avoidance behavior to “bitter-tasting” irritant compounds

**Figure 3.**
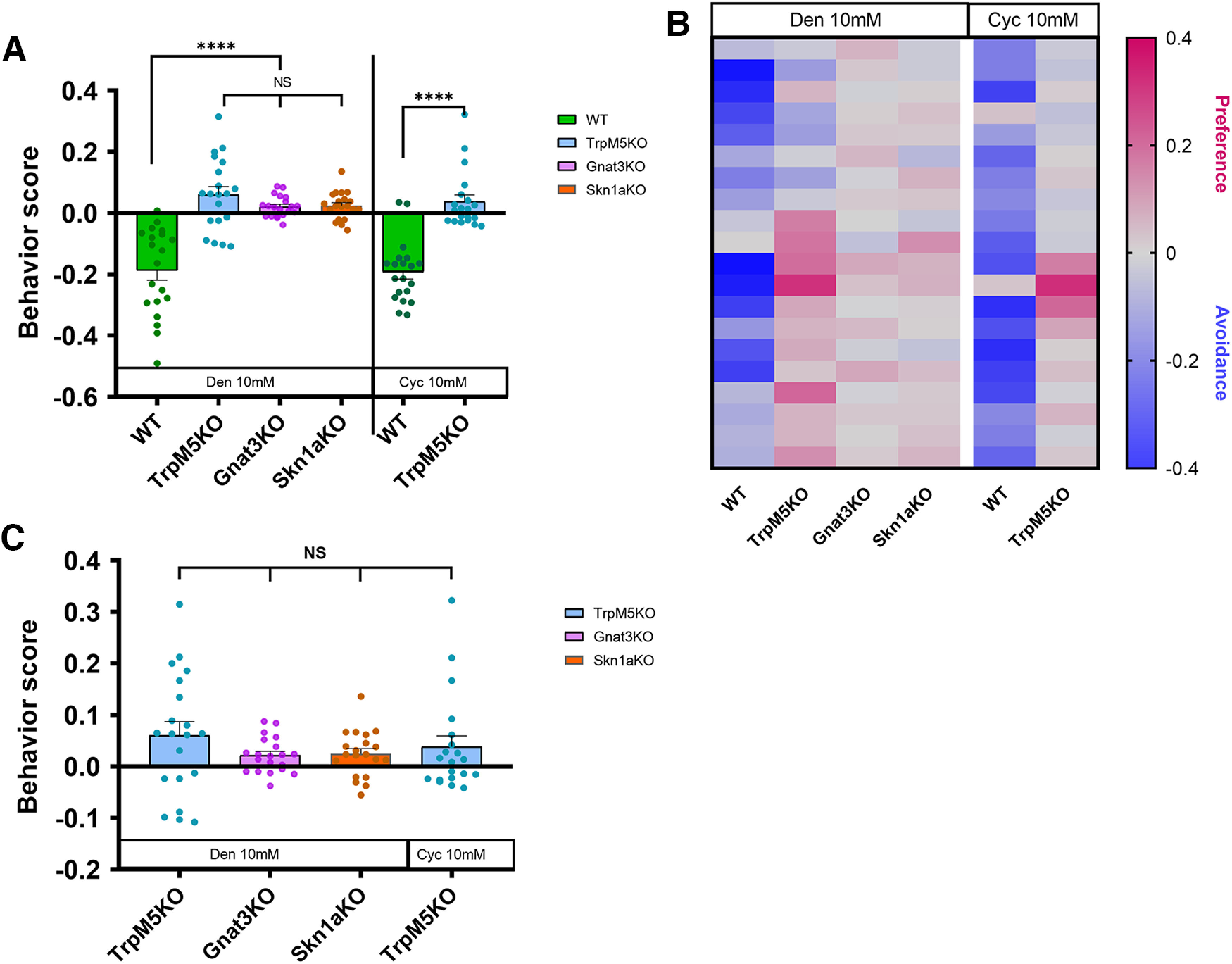
Avoidance behavior to bitter compounds in wild-type (WT) mice and solitary chemosensory cell (SCC) pathway knock-out (KO) mice. ***A***, Naive WT mice exposed to denatonium (Den) and cycloheximide (Cyc) exhibited avoidance responses, spending ∼70% of the time in the saline chamber. In contrast, the three KO mice showed no avoidance but instead a certain degree of interest in the irritant-containing chamber. *n* = 10 mice per group, 60 mice in total. Behavioral scores below 0 indicate avoidance of irritants and above 0 indicate attraction. ****p ≤ 0.0001, NS: no significance. Bars and symbols reflect mean ± SEM. ***B***, Heat map representation of behavior score for each mouse for the data in ***A***. Each rectangle represents the ratio of time a mouse spent in the left versus right chamber. Blue and red represent an increase in avoidance and preference, respectively, with color tones indicating intensity; gray is the baseline (behavior score = 0) representing indifference (see Extended Data Fig. 3-1 for supporting information/data). ***C***, Representation of the data analysis of just the KO mice stimulated with either Den or Cyc from ***A***. All KO mice exposed to either Den or Cyc show nonstatistical differences between each other in their behavioral score. NS: no significance. Bars and symbols reflect mean ± SEM.

During the first session of the avoidance assay for control experiments, when both chambers contained nebulized saline, WT and KO mice showed no avoidance or preference, spending equal time in both chambers (Extended Data Fig. 3-1). For the avoidance behavior assay experiments, after a few brief explorations of the two chambers, WT mice learned quickly to avoid the chamber containing Den or Cyc, and settled in the saline chamber, while showing signs of discomfort, respiratory reflexes, and freeze behavior. Naive WT mice exposed to 10 mm Den or 10 mm Cyc spent statistically much more time (around 70% of the entire time) in the chamber containing nebulized saline solution (WT represented in green; one-way ANOVA followed by Tukey’s *post hoc* test, *p* < 0.0001; Fig. 3*A*). In contrast, KO mice missing elements of the SCC signaling cascade (TrpM5-KO and Gnat3-KO) or missing SCCs (Skn1a-KO) spent 36–46% of the time in the saline chamber, with no statistical preference for the chamber containing Den (all three KOs) or Cyc (TrpM5-KO; Fig. 3*A*,*C*, with *C* representing the data analysis of just the KO mice stimulated with either Den or Cyc from *A*; one-way ANOVA followed by Tukey’s *post hoc* test, *p*>0.99). During the experiments, mice never licked their fur and/or the chamber surfaces (Extended Data 1). The heat map in Figure 3*B* represents the second and third 5-min recording sessions for each mouse (*n* = 10), with red and blue representing preference and avoidance for the Den/Cyc, filled chamber, respectively. The absence of avoidance behavior and protective reflexes that drive the avoidance response to Den or Cyc in KO mice suggests that functional SCCs and activation of SCCs by Den or Cyc are necessary for the avoidance response.

10.1523/ENEURO.0245-22.2023.f3-1 Extended Data Figure 3-1 Heat map representation of saline versus saline behavioral responses for all mice used in these experiments. ***A***, Heat map of behavior scores for the 40 wild-type (WT) and knock-out (KO) mice exposed to saline in both chambers used for the olfactory epithelium ablation experiments with 10 mm denatonium (Den). ***B***, Heat map for the 140 WT and KO mice exposed to saline in both chambers used for the dose-response (2, 5, and 10 mm Den) and repeated-exposure experiments (10 mm Den and 10 mm cycloheximide). Each rectangle represents the ratio of time the mouse spent in the left versus right chamber. Blue and red represent an increase in avoidance and preference, respectively, with color tones indicating intensity; gray is the baseline (behavior score = 0), representing indifference. Download Figure 3-1, TIF file.

### Avoidance behaviors positively correlate with denatonium concentration

**Figure 4.**
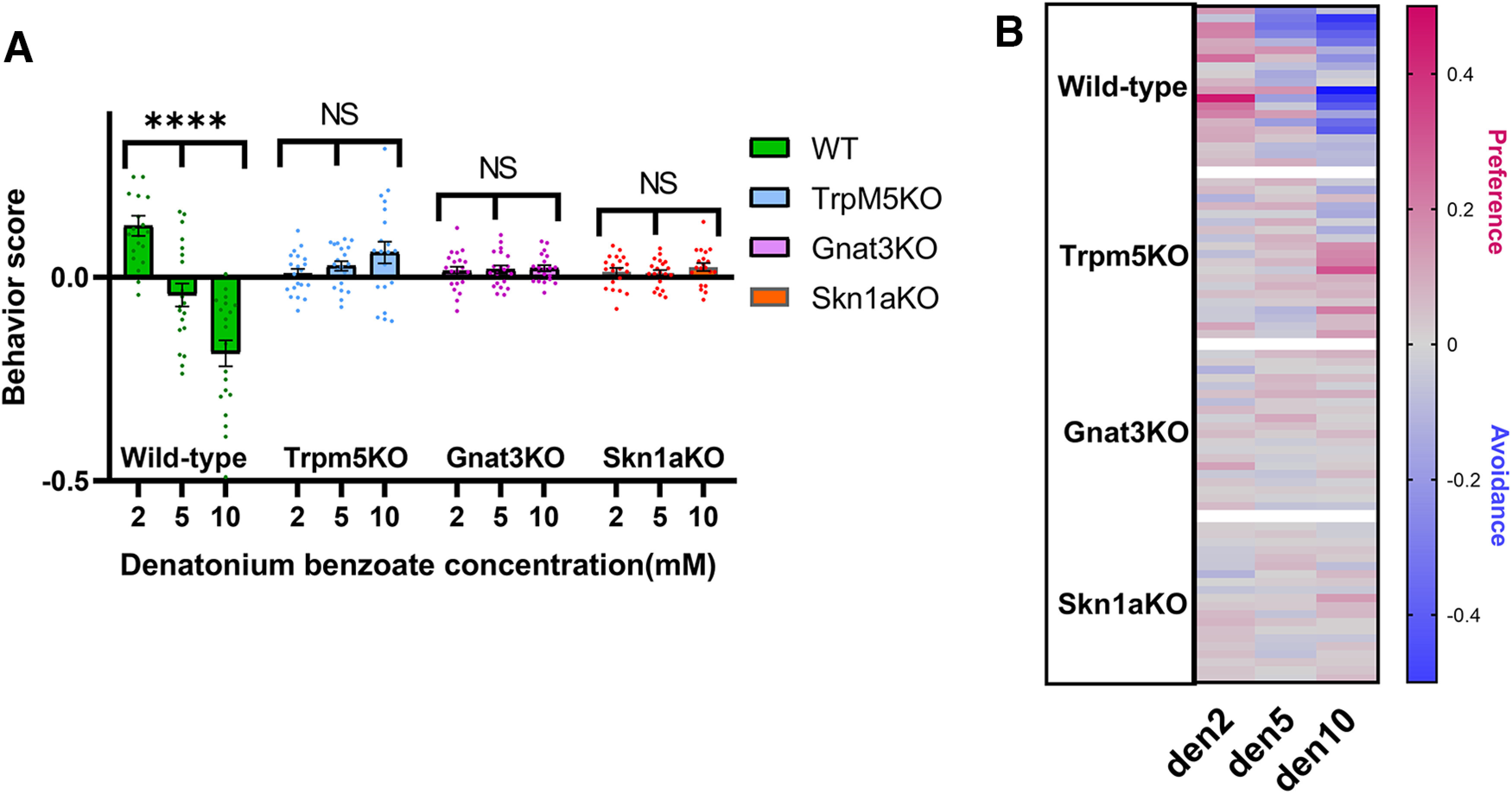
Dose-dependent avoidance behavior to denatonium (Den) in naive wild-type (WT) and solitary chemosensory cell (SCC) pathway knock-out (KO) mice. ***A***, Avoidance behavior increased in WT mice as the Den concentration increased. WT mice preferred 2 mm Den (den2) and avoided 10 mm Den (den10). TrpM5-KO, Gnat3-KO, and Skn1a-KO mice spent equal time in the irritant and saline aerosolized chambers, showing no significant difference at the three Den concentrations. *n* = 10 mice per group, 120 mice in total. Behavioral scores below 0 indicate avoidance of irritants and above 0 indicate attraction. ****p ≤ 0.0001, NS: no significance. Bars and symbols reflect mean ± SEM. ***B***, Heat map representation of behavior score for each mouse for the data in ***A***. Each rectangle represents the ratio of time the mouse spent in the left versus right chambers. Blue and red represent an increase in avoidance and preference, respectively, with color tones indicating intensity; gray is the baseline (behavior score = 0) representing indifference.

Naive WT mice responded to nebulized Den in a dose-dependent manner. When exposed to 2, 5, or 10 mm Den, WT mice exhibited a preference at 2 mm Den, a neutral behavior at 5 mm, and a marked avoidance response at 10 mm (two-way ANOVA followed by Tukey’s *post hoc* test; Fig. 4*A*). The fact that WT mice do not avoid nebulized Den at 2 mm is surprising considering that mice avoid drinking Den solution starting at 0.1 mm because of its bitter taste (Finger et al., 2005). It is difficult to make a correlation between chemicals delivered in the atomized form through the nose or dissolved in the mucus that plausibly reaches the tongue through the nasal cavity. Thus, although Den is delivered at 2 mm concentration as an aerosol into the nose, the threshold concentration that, presumably, reaches the tongue is unknown and, possibly, not enough to activate the bitter taste receptors. While we cannot imply that the taste system is not involved in the avoidance response with this experiment, we can speculate that as observed during the avoidance assay experiments mice are, probably, not getting any Den activating the taste system from either liking the mouse body hairs or direct contact with the chamber surfaces, and if any Den reaches the tongue, it would be retronasally at an unknown concentration. Another interesting observation in this experiment was that WT showed a preference instead of avoiding the chamber nebulized with 2 mm Den, suggesting that mice possibly used their sense of smell to detect Den and explored the chamber containing Den because it “smells like something” while the saline nebulized chamber had “no smell.” Coppola and colleagues reported that naive WT mice can smell Den as well as other bitterants, and mice with a bilateral bulbectomy do not learn or retain the ability to avoid Den vapor, implicating olfaction as the mode of detection (Coppola and Slotnick, 2018). We confirmed in this experiment that WT mice avoid 10 mm Den vapor in line with previous Ca-imaging experiments where SCCs detect Den starting at 10 mm (Gulbransen et al., 2008; Tizzano et al., 2010; Saunders et al., 2014), suggesting a primary role for the nasal SCCs in the avoidance behavior to Den.

In contrast to WT, all the KO mice (TrpM5-KO, Gnat3-KO, and Skn1a-KO) lacked any significant differences in avoidance responses to 2, 5, and 10 mm Den (Fig. 4*A*). Naive KO mice did not show any statistically measurable preference behavior at all three Den concentrations (Fig. 4*A*). The TrpM5-KO, Gnat3-KO, and Skn1a-KO mice are missing elements or cells of the taste chemosensory pathway in several mucosae. In the MOE, MCs participate to maintaining the olfactory function and the lack of attraction to Den may be attributable to these chemosensory cells, thus their function may assist the OSNs in the detection of this compound. The heat map in Figure 4*B* represents the second and third 5-min recording sessions for each mouse (*n* = 10), with red and blue representing preference or avoidance for the Den-filled chamber, respectively. These data support the ideas that the avoidance behavior positively correlates with Den concentration and that an intact SCC signaling pathway is necessary for the avoidance response.

### Improved avoidance in wild-type and preference in knock-out naive mice after repeated exposures to denatonium

**Figure 5.**
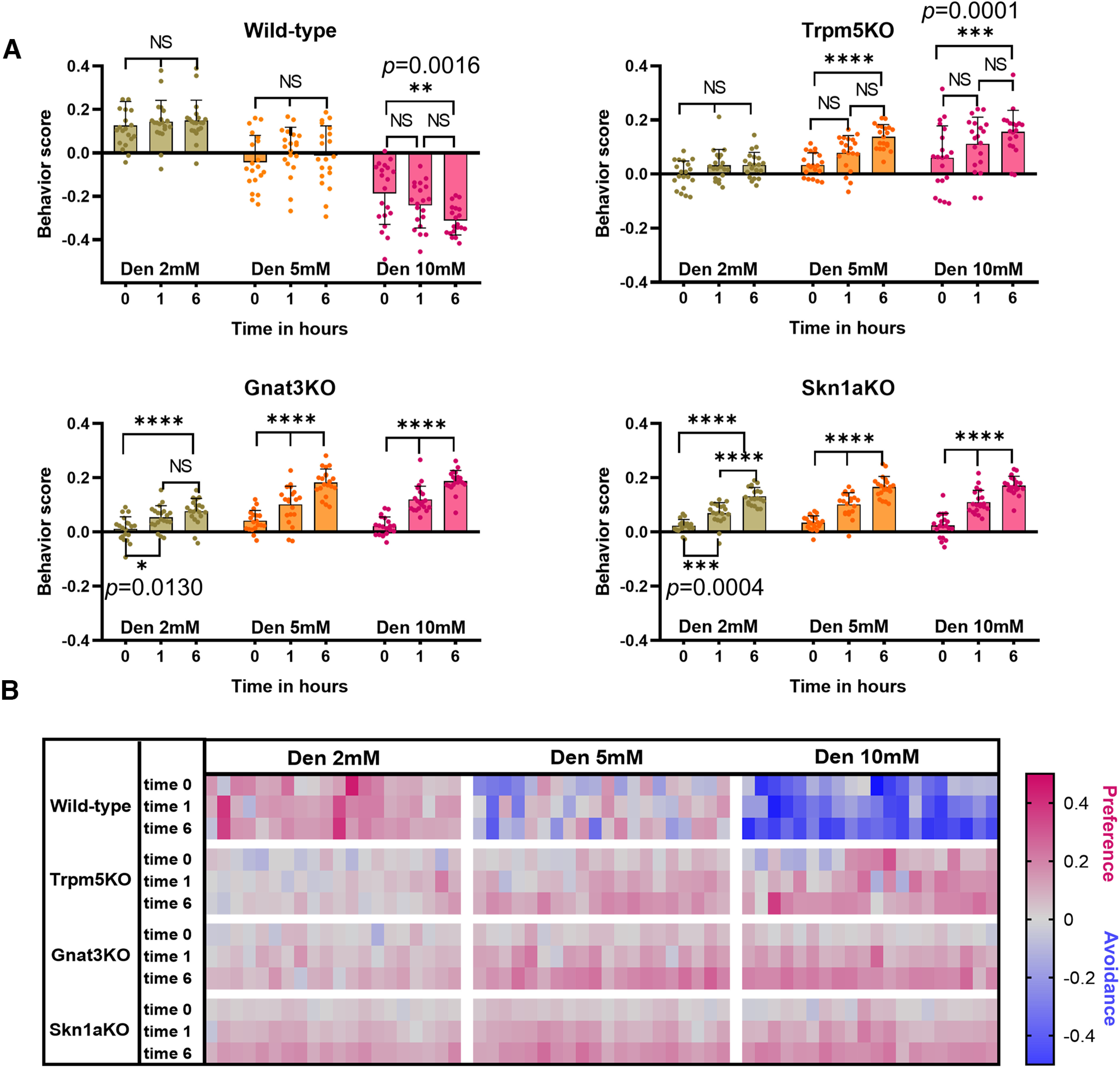
Dose-dependent avoidance behavior after repeated exposure to denatonium (Den) in wild-type (WT) and solitary chemosensory cell (SCC) pathway knock-out (KO) mice. ***A***, Avoidance in WT mice and preference in KO mice after the first naive exposure to Den improved after repeated exposures to all Den concentrations. WT mice showed faster avoidance responses for 10 mm but not for other concentrations of Den directly proportional to the number of repeated exposures. The KO mice also showed a significant improvement in preference behavior after repeated exposure either to Den 5 and 10 mm (TrpM5-KO) or to 2, 5, and 10 mm (Gnat3-KO and Skn1a-KO; see Extended Data Fig. 5-1 for supporting information/data). *n* = 10 mice per group, 120 mice in total. Behavioral scores below 0 indicate avoidance of irritants and above 0 indicate attraction. *0.01 < *p* ≤ 0.05, **0.001 < *p* ≤ 0.01, ***0.0001 < *p* ≤ 0.001, ****p ≤ 0.0001, NS: no significance. Bars and symbols reflect mean ± SEM. ***B***, Heat map representation of behavior score for each mouse for the data in ***A***. Each rectangle represents the ratio of time the mouse spent in the left versus right chambers. Blue and red represent an increase in avoidance and preference, respectively, with color tones indicating intensity; gray is the baseline (behavior score = 0), representing indifference.

The avoidance response in WT mice and the preference response in TrpM5-KO, Gnat3-KO, and Skn1a-KO mice improve significantly after repeated exposure to nebulized Den (Fig. 5). Naive WT mice exposed to 2 and 5 mm Den (time 0) showed no significant changes in the time spent in saline versus Den chamber during following exposures (times 1 and 6 h; Fig. 5*A*, top left). At 10 mm Den, the concentration that activates nasal SCCs, the avoidance response observed in naive WT mice was faster with significantly less time spent in the Den-filled chamber at time 6 h than at time 0 (two-way ANOVA followed by Tukey’s *post hoc* test; Fig. 5*A*, top left). The average time naive WT mice spent in the 10 mm Den chamber was 31.2%, which decreased to 25.8% and 18.8% at the second and third exposures (*p* = 0.0016). The Gnat3-KO and Skn1a-KO mice significantly improved their preference behavior after the first exposure to all concentrations of Den and spent more time in the Den-filled chamber (*p* < 0.0001; Fig. 5*A*, bottom), while the TrpM5-KO mice showed a significantly improved preference response only at 5 and 10 mm Den and at the 6-h time point (Fig. 5*A*, top right). Interestingly, at 2 mm Den only the WT but not the KO mice shows a consistent preference for Den at all three time points (0,1, and 6 h), with the KO mice learning after the first exposure to recognize Den using other clues than the SCC pathway (probably the smell of Den) and improving their preference for Den similarly to the WT mice (Extended Data Fig. 5-1). Both data in Figures 4*A* and 5*A* support the possible involvement of MCs in the preference behavior for 2–10 mm Den in naive mice. Upon repeated exposure to all three Den concentrations, other epithelial cells contribute to the preference response in the TrpM5-KO, Gnat3-KO, and Skn1a-KO mice. The absence of MCs in these KO mice suggests that MCs may be involved in the preference response to naive presentation of Den observed in WT mice, while the OSNs contribute by improving the preference behavior responses after repeated Den exposure in the KO mice and increase the avoidance response in the WT mice (Fig. 5*A*). The heat map (Fig. 5*B*) represents the second and third 5-min recording sessions for each mouse (*n* = 10) at each time point (0, 1, and 6 h), with red and blue colors representing preference and avoidance for the Den-filled chamber, respectively. These data show that the avoidance and preference responses in WT and KO mice positively correlate with the Den concentration and the number of subsequent exposures, indicating that mice can learn to identify Den more quickly with the assistance of other sensory systems, like taste and smell, that contribute to the behavioral responses after repeated exposure to Den.

10.1523/ENEURO.0245-22.2023.f5-1 Extended Data Figure 5-1 Preference for 2 mm Den in both WT and SCC-KO mice. Both WT and Gnat3-KO, TrpM5-KO, and Skn1a-KO mice showed a consistent preference and/or improved behavior to 2 mm Den at all three time points (0, 1, and 6 h). *n* = 10 mice per group, 40 mice in total. Behavioral scores below 0 indicate avoidance of irritants and above 0 indicate attraction/preference. *0.01 < *p* ≤ 0.05, **0.001 < *p* ≤ 0.01, ***0.0001 < *p* ≤ 0.001, ****p < 0.0001, NS: no significance. Bars and symbols reflect mean ± SEM. Download Figure 5-1, TIF file.

### Sensory input from the olfactory but not the taste system contributes to the behavior responses

WT mice can taste and avoid Den at 0.1–1 mm concentrations (Finger et al., 2005), while nasal SCCs respond to 10 mm or higher concentrations of Den (Gulbransen et al., 2008; Tizzano et al., 2010; Saunders et al., 2014). Thus, a mouse should be able to detect the bad “bitter” taste of 2 mm Den. However, in our avoidance assay experiments, WT mice did not avoid but showed a preference behavior for 2 mm Den (Fig. 4). Moreover, mice during the avoidance assays do not perform any licking of their fur or of the chamber surfaces (Extended Data 1). Altogether, the evidence suggests that the taste system is probably not involved in the behavioral response to vaporized Den at this concentration. In TrpM5-KO, Gnat3-KO, or Skn1a-KO mice, that lack both the SCC and taste pathways, the avoidance response to 2–10 mm Den was missing, implying that functional SCCs and activation of SCCs by Den are necessary for the avoidance behavior, but not providing conclusive evidence that would exclude the participation of the taste system in the avoidance behavior. As discussed above, the KO mice showed a preference behavior for the chamber nebulized with Den, probably based on input from a sensory system other than SCCs and taste.

**Figure 6.**
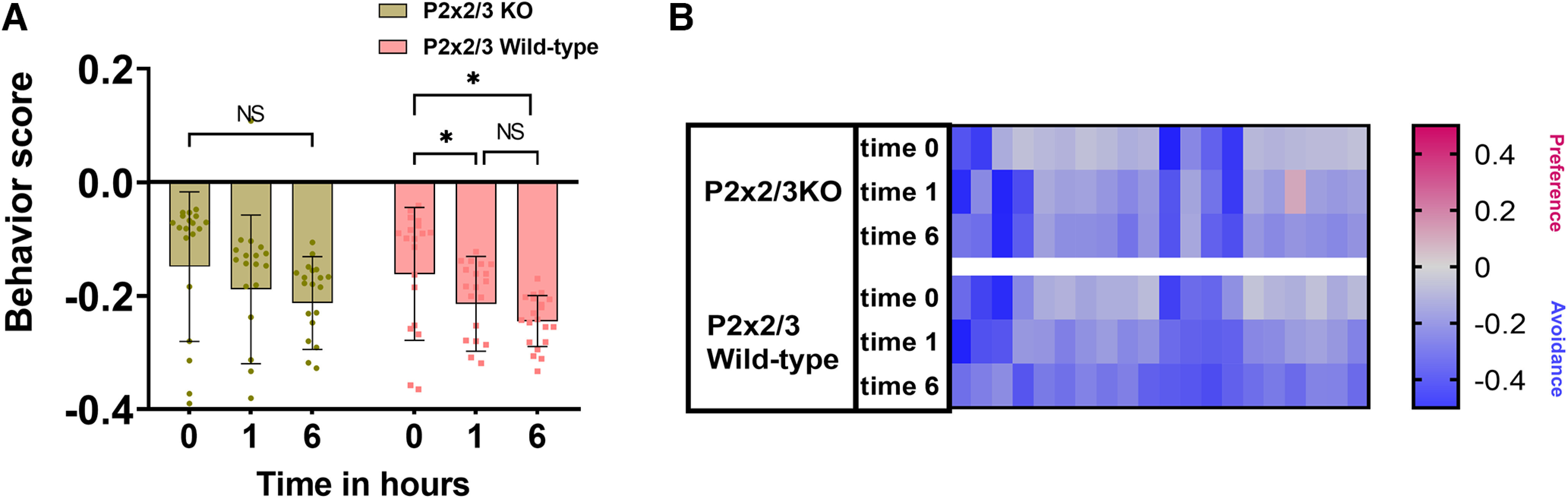
Sensory input from the taste system does not contribute to the behavior responses. ***A***, Bitter-ageusic transgenic P2X2/3-double-KO mice still avoid 10 mm Den, implying that SCCs but not the taste system are involved in the avoidance behavior response to nebulized irritants. Both P2X2/3-double-KO and wild-type mice respond similarly to Den at all time points. *n* = 10 mice per group, 20 mice in total. Behavioral scores below 0 indicate avoidance of irritants and above 0 indicate attraction. NS: no significance. Bars and symbols reflect mean ± SEM. ***B***, Heat map representation of behavior score for each mouse for the data in ***A***. Each rectangle represents the ratio of time mice spent in the left versus right chambers. Blue and red represent an increase in avoidance and preference, respectively, with color tones indicating intensity; gray is the baseline (behavior score = 0) representing indifference.

To determine the role of the taste system in the behavioral responses to Den, we used the bitter-ageusic transgenic P2X2/3-double-KO mouse, which is missing a functional taste system. The P2X2/3-double-KO mouse avoids 10 mm Den similarly to the control WT (Fig. 6*A*). When naive or exposed repeatedly to 10 mm Den, both P2X2/3-double-KO and WT mice showed no statistical differences in their avoidance behavior. Like in previous experiments (Den 10 mm; Fig. 5*A*, top left), both P2X2/3-double-KO and wild-type mice seem to perform better in subsequent time point exposures (1 and 6 h; Fig. 6*A*). The heat map in Figure 6*B* represents recording sessions for each mouse (*n* = 10), with red and blue representing preference or avoidance for the Den-filled chamber, respectively. These data support the idea that the taste system is not involved in the avoidance behavioral response to Den and that the SCC signaling pathway is mainly responsible for the avoidance response.

**Figure 7.**
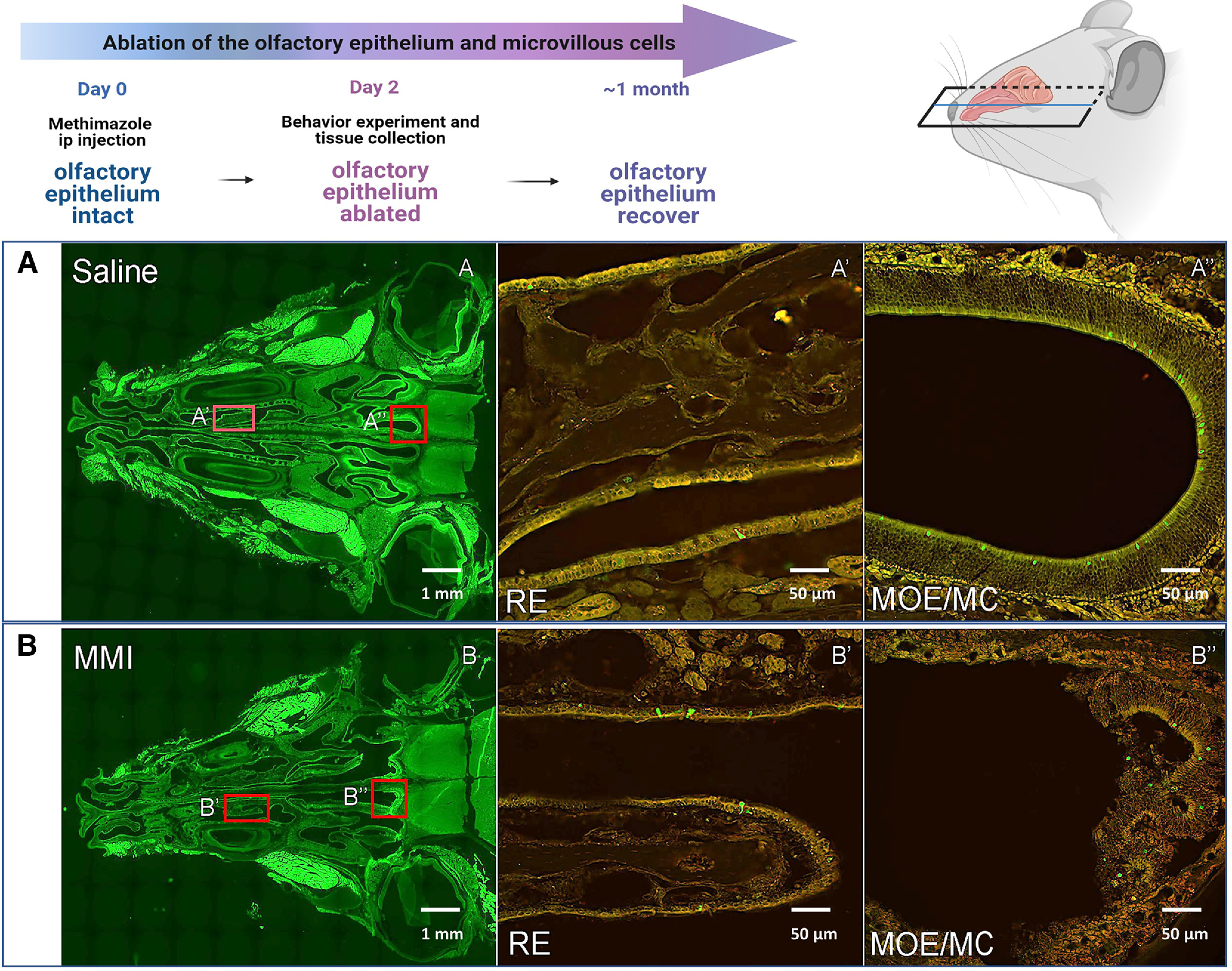
Ablation of the olfactory sensory epithelium after methimazole injection (MMI). Top, MMI experiment timeline and schematic representation for the sectioning orientation of the TrpM5-GFP mice with MMI and saline injection. Mice were injected at day 0 where the olfactory epithelium is still intact. Two days after MMI, mice were used for the avoidance behavior experiments. TrpM5-GFP mice injected with MMI were euthanized after 2 d, and tissue was collected for immunohistochemical examination to show the ablation of the olfactory epithelium and consecutive smell loss. ***A***, Saline-injected mice showed normal respiratory (RE) and main olfactory epithelium (MOE). ***A’***, ***A’’*** are magnifications of the red insets in ***A*** showing intact RE and the presence of TrpM5-GFP-positive solitary chemosensory cells (SCCs) in the RE and green fluorescent protein (GFP)-positive microvillous cells (MCs) in the MOE. ***B***, After 2 d, MMI mice showed a normal RE (***B’***) with healthy GFP-positive SCCs, but the MOE (***B’’***) was detached from the basal lamina and is nonfunctional, resulting in the loss of the sense of smell. In the MOE, GFP-positive MCs are still visible, crumpled at the back of the turbinate, but this tissue is not alive and will disappear in a few days.

**Figure 8.**
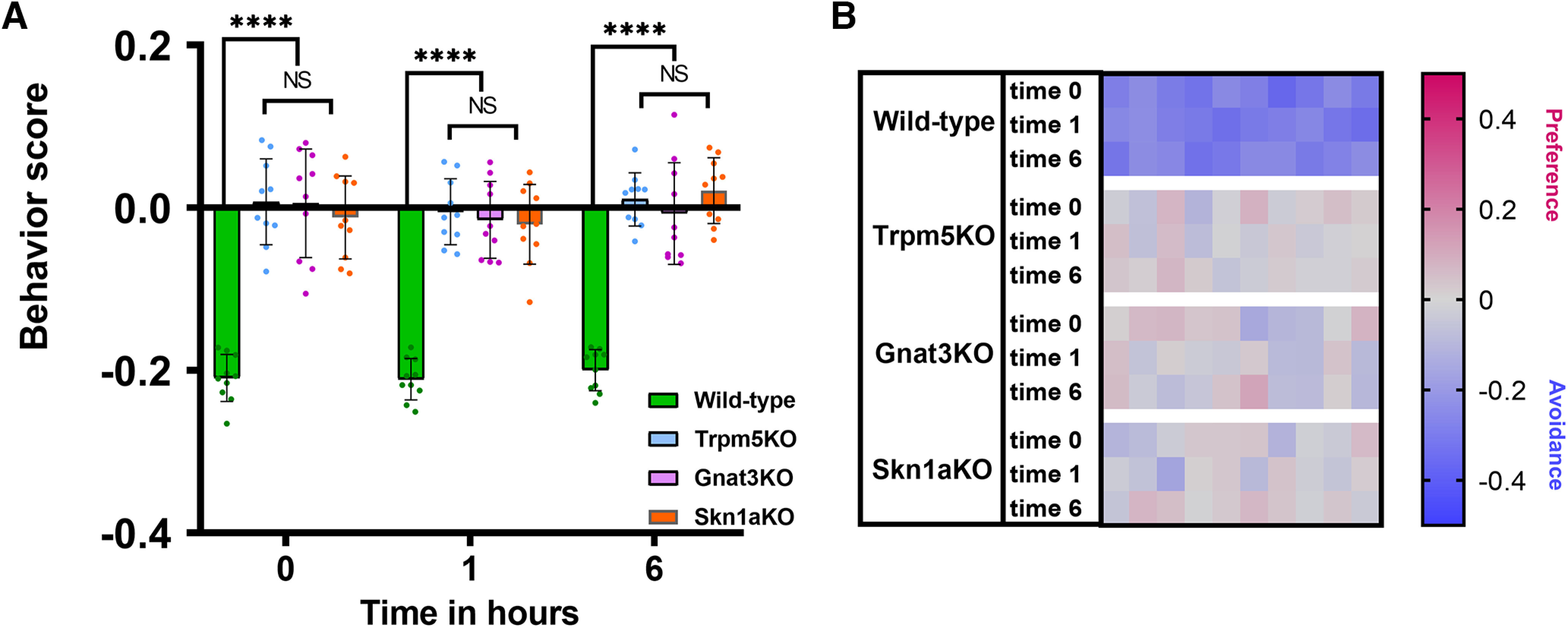
Sensory input from the olfactory system contributes to the behavior responses. Mice treated with methimazole lose the sense of smell and lack the improvement of avoidance or preference behavioral responses present in untreated control mice. ***A***, After methimazole injection, the significant improvement in the avoidance response to 10 mm Den (Fig. 5*A*) shown by the knock-out (KO) mice was missing in WT mice at all time points (see Extended Data Fig. 8-1 for supporting information/data). TrpM5-KO, Gnat3-KO, or Skn1a-KO mice injected with methimazole did not show preference behavior to 10 mm Den. *n* = 10 mice per group, 40 mice in total. Behavioral scores below 0 indicate avoidance of the irritant and above 0 indicate attraction. ****p ≤ 0.0001, NS: no significance. Bars and symbols reflect mean ± SEM. ***B***, Heat map representation of behavior score for each mouse for the data in ***A***. Each rectangle represents the ratio of time mice spent in the left versus right chambers. Blue and red represent an increase in avoidance and preference, respectively, with color tones indicating intensity; gray is the baseline (behavior score = 0) representing indifference.

To test whether the olfactory system contributes to behavioral responses preferences, we pharmacologically ablated the olfactory epithelium (OE) and microvillous cells (MVCs; SCC-like cells located superficially on the OE) with a methimazole injection (MMI) that causes the detachment of several cell types in the OE from the basal lamina and consequent loss of the olfactory function (Fig. 7). Two days post-MMI, TrpM5-GFP-positive SCCs and the respiratory epithelium (RE) are still preserved (Fig. 7*B’*) and look anatomically no different from in untreated mice (Fig. 7*A’*). MMI treatment caused detachment of the OE from the basal lamina, and only a few pieces of dead OE with few decaying GFP-positive MVCs can be seen around the nasal turbinates (Fig. 7*B’’*). For the avoidance behavior assay, WT and TrpM5-KO, Gnat3-KO, and Skn1a-KO mice received MMI and, after 2 d, were tested for behavioral responses to 10 mm Den. WT mice avoided Den at 0-, 1-, and 6-h time points (two-way ANOVA followed by Tukey’s *post hoc* test; Fig. 8*A*), showing that the RE is still intact and the SCC pathway is fully functional. Interestingly, the significant improvement in the WT avoidance response to 10 mm Den observed at the 6-h time point (Fig. 5*A*) was missing in the WT+MMI group (Extended Data Fig. 8-1*A*, red arrow), showing that WT mice use the sense of smell to recognize Den and learn to avoid it more quickly after multiple exposures, spending less time in the Den-filled chamber. TrpM5-KO, Gnat3-KO, and Skn1a-KO mice with MMI lost the preference behavior to 10 mm Den (two-way ANOVA followed by Tukey’s *post hoc* test; Fig. 8*A*) or the quicker preference responses (Extended Data Fig. 8-1*B–D*, red arrows) that the KO mice showed before MMI. This confirms that the KO mice can smell Den and probably are attracted to the smell of the Den-filled chamber in the absence of the sensory discomfort mediated by SCCs, which are nonfunctional in these KO mice. Moreover, local hyperalgesic responses to Den were not observed in either WT nor KO MMI treated mice, as underlined by the absence of any increased avoidance or preference in MMI mice (Extended Data Fig. 8-1, red arrows). We believe that the nasal epithelium injuries caused by methimazole ablation of the OE are surely triggering inflammatory reactions, but this seems not to affect the behavior assay responses or cause hyper-responsiveness of the nasal epithelium. The heat map in Figure 8*B* represents the second and third 5-min recording sessions for each mouse (*n* = 10), with red and blue representing preference or avoidance for the Den-filled chamber, respectively.

10.1523/ENEURO.0245-22.2023.f8-1 Extended Data Figure 8-1 Methimazole experiment data for mice injected with methimazole for each mouse strain and at each time point. After ablation of the sense of smell, both WT and KO mice lost the quicker avoidance and preference responses after methimazole injection (red arrows). *n* = 10 mice per group, 40 mice in total. Behavioral scores below 0 indicate avoidance of irritant and above 0 indicate attraction. **0.001 < *p* ≤ 0.01, ****p < 0.0001, NS: no significance. Bars and symbols reflect mean ± SEM. Download Figure 8-1, TIF file.

## Discussion

**Figure 9.**
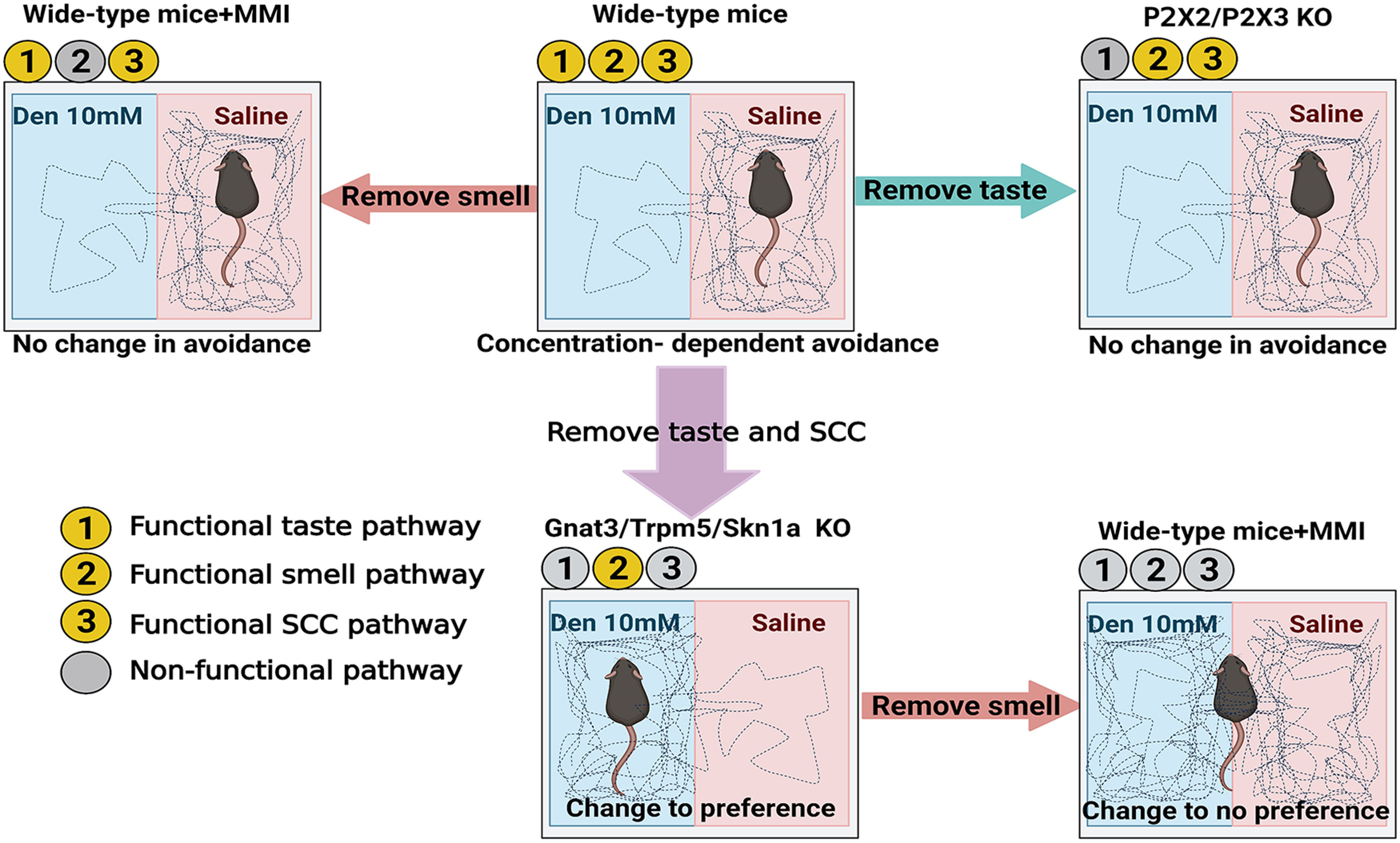
Summary schematic illustration of the role of solitary chemosensory cells (SCCs) in avoidance behavior to aerosolized irritants. Wild-type (WT) mice with intact taste, smell and SCC pathways avoid denatonium (Den) in a concentration-dependent manner that improves when experiencing multiple exposures to Den. Knock-outs of key elements of the SCC signaling pathway (Gnat3-KO and TrpM5-KO) or lack of SCCs (Skn1a-KO) but that also lack taste show a behavioral preference to Den probably because Den become attractive in the absence of discomfort and pain mediated in WT by the SCC pathway. Mice without a functional taste system (P2X2/P2X3 double KO) demonstrate that SCCs but not taste participates in the avoidance behavior to nebulized Den. To determine the role of the olfactory system in the behavior responses to nebulized Den, WT, and KO mice were injected with methimazole (MMI = methimazole injection) that induces temporary loss of smell. In WT mice with MMI, the avoidance behavior to Den was still observed although the faster avoidance response evoked by repeated exposure was eliminated, demonstrating that, even without the sense of smell, WT mice can detect and avoid Den and that the olfactory system contributes to the learning of a quicker avoidance of Den. In TrpM5-KO, Gnat3-KO, and Skn1a-KO mice with MMI, the faster Den preference responses disappear demonstrating that also in this case the learning portion of the response is mediated by the sense of smell.

In our experiments, the avoidance behavior observed in naive mice exposed to inhaled Den and Cyc depended on the activation of the SCCs, rather than the taste system or the smell system, as confirmed using mouse models missing functional taste, smell, or SCC pathways (Fig. 9). WT mice avoided these irritants likely by the discomfort evoked after the SCC activation triggers protective reflex responses (e.g., sneezing, apnea, laryngospasms, and expiration reflexes). In the absence of SCCs and protective reflexes mediated by the SCCs, the avoidance responses to Den and Cyc in TrpM5-KO, Gnat3-KO, and Skn1a-KO mice were lost, showing the importance and implication of these nasal epithelial cells as sensory sentinels for irritants (Fig. 9). Because Den is an extremely bitter compound, WT mice will avoid drinking it at concentrations starting from 0.1 to 1 mm (Finger et al., 2005). Surprisingly, WT mice showed a preference for Den at 2 mm despite the Den bitter taste. We can only speculate that avoidance at 2 mm Den exposure in WT mice is absent because Den does not reach the threshold concentration in the saliva to stimulate bitter taste receptors when the nebulized solution reaches the tongue, presumably through the nasal cavity. It is plausible that the avoidance observed at 10 mm Den occurs because Den would reach the tongue at a concentration high enough to activate the bitter taste system. Moreover, during the avoidance assay, we did not observe any grooming behavior or licking of the chamber surfaces, confirming that the taste system cannot be reached or activated by direct oral exposure (Extended Data 1). To better understand whether the taste, the SCCs, or both sensory systems are involved in the avoidance behavior to 10 mm Den in WT mice, we used bitter-ageusic P2X2/X3 double knock-out mouse model, which lacks purinergic receptors involved in the sweet, bitter, and umami taste signaling transduction. Similar to WT mice, the P2X2/X3 double KO mice avoided Den, confirming that the taste system is not involved in the avoidance behavior. Thus, mice are not simply avoiding a chamber that “tastes bad” (Fig. 9) but are instead avoiding the chamber in response to the activation of the nasal SCCs by nebulized Den.

After naive animals were repeatedly exposed to the irritant, both WT and SCC-KO mice reinforced their respective avoidance or preference behaviors demonstrating that they can learn from multiple sensory inputs to behave more quickly and sharply to the irritant exposure. In the case of WT mice, this reinforced behavior was the result of the SCC-mediated aversive response plus a contribution from the olfactory system presumably by MCs and OSNs (i.e., the irritant’s “odor”) resulting in a quicker and more effective avoidance of the irritant. In the SCC-KO mice, in the absence of the SCC-mediated protective reflexes, the preference response was mostly triggered by the odor of the irritant. In the TrpM5-KO, Gnat3-KO, and Skn1a-KO mice, MCs were missing, or the chemosensory pathway was disrupted. It is plausible that the preference responses to 2–10 mm Den observed in naive WT but absent in all the KO mice is attributable to MCs, that assist in maintaining the olfactory function of the MOE (Lemons et al., 2017). Upon multiple exposure to Den, TrpM5-KO, Gnat3-KO, and Skn1a-KO mice all showed a statistical increase in the preference behavior suggesting that other epithelial elements, seemingly the OSNs, are involved in the response. Using targeted pharmacological disruption of the olfactory sensory epithelium (Fig. 9), we validated the involvement of olfaction in the behavior responses, and we have shown that mice learn to perform better when the stimulus was presented more than once. In WT mice, we demonstrated that the improved avoidance behavior consists of at least two components, the olfactory response component derived from multiple Den exposures, at 0, 1, and 6 h, that disappears in mice injected with methimazole, and the remaining avoidance response component that represents the SCC-mediated component of the behavior that is independent from the olfactory component and remain constant at all three time points (Fig. 8*A*; Extended Data Fig. 8-1*A*). Furthermore, mice in which both the SCC system (TrpM5-KO, Gnat3-KO, and Skn1a-KO), MCs, and the olfactory system (methimazole-induced anosmia) were disrupted, showed no difference in response to Den, highlighting and validating the partial involvement of the sense of smell in the avoidance behavior to nebulized Den (Fig. 8*A*; Extended Data Fig. 8-1*B–D*).

The observations in this study determining the involvement of the SCC sensory system in the avoidance behavior to nebulized Den and Cyc are novel and consistent with previous studies that involve the stimulation of the trigeminal sensory system with ammonia, ozone, formalin, acrolein, toluene, acetic acid, formaldehyde, and other natural or man-made chemicals. A few of these studies reported physiological and behavioral responses to inhaled irritants: breathing pattern depression, increased respiratory-specific airway flow resistance, anxiety-like behavior, decreased respiratory frequency, and “escape behavior” from the irritant (Tepper and Wood, 1985; Wood and Coleman, 1995; Bryant, 2000; Morris et al., 2003; Saunders, et al., 2013; Yonemitsu et al., 2013; Kim et al., 2020). Only one study reported on enhanced avoidance response sensitivity associated with repeated exposure (Wood and Coleman, 1995), and another described using a chamber assay to study escape behavior from a contaminated environment (Yonemitsu et al., 2013).

In mice, olfaction and the trigeminal system play pivotal roles in various behaviors, such as feeding, mating, nursing, and avoidance. Few behavioral tests exist to investigate the abilities of odor detection, recognition, and avoidance in mice (Takahashi and Tsuboi, 2017). Very little is known about mouse behavioral responses to inhaled irritants, specifically to compounds activating protective reflexes through the SCC signaling pathway. Our dual-chamber forced-choice device provides a tool to study avoidance and attraction behaviors to nebulized chemicals. Moreover, the avoidance behavior assay we developed requires only a few minutes for each session to determine whether mice avoid a chemical, how much they avoid it, and whether they avoid it more efficiently after repeated exposure.

The present study is the first report of an avoidance function by the SCC sensory system to a certain class of chemical compounds that includes bitter tastants (Den and Cyc) and bacterial metabolites (Cyc). Our results demonstrated the primary involvement of the SCCs in avoidance responses to nebulized irritants and identify the contributions of other sensory systems to the behavior. Certain inhaled irritants may have an agreeable smell and elicit no acute noxious sensations at lower concentrations, so their avoidance behavior must be learned using other sensory clues. We have no knowledge of past studies looking at how different sensory systems coexist and contribute to behavioral responses in mice. Our behavioral protocol and the KO mice used in these studies provide answers on the involvement of each sensory system and its contribution to the innate and learned avoidance behavior responses to inhaled irritants.

The environment in which we live contains many harmful compounds and xenobiotics that can be inhaled and that can assault the nasal cavity. Consequences of such inhalations can range from simple nasal congestion and inflammation to permanent airway damage or even death if we fail to detect a lethal compound. Nasal detection of harmful compounds is historically known to be mediated by the trigeminal chemosensory system. Research produced in the last two decades, including from our group, has shown that the nasal SCC sensory system aids in detecting harmful inhaled compounds, evoking inflammatory and immune responses and respiratory-protective reflexes to eliminate and prevent further inhalation of these compounds. The SCC-mediated avoidance behavior reported in detail here represents an important defense mechanism against the inhalation of noxious chemicals to minimize mucosal damage. Together, the trigeminal and SCC sensory systems, assisted by the olfactory system, represent powerful layers of protection from noxious chemical inhalation and respiratory damage.

10.1523/ENEURO.0245-22.2023.ext1 Extended Data 1 Zip file containing the MATLAB code, an example video used to track the behavior, and the example video showing typical behavior during a recording session. Download Extended Data 1, ZIP file.

## Synthesis

Reviewing Editor: Matthew Grubb, King’s College London Decisions are customarily a result of the Reviewing Editor and the peer reviewers coming together and discussing their recommendations until a consensus is reached. When revisions are invited, a fact-based synthesis statement explaining their decision and outlining what is needed to prepare a revision will be listed below. The following reviewer(s) agreed to reveal their identity: Ivan Manzini, Roberto Tirindelli.

Reviewer 1:

The authors have addressed all my points and answered all my questions. The additional experiments have convinced me. Also, the authors now better discuss their results. The revised version of the manuscript has been substantially revised and improved. I want to commend the authors for their effort.

It is an excellent and exciting study.

I only have a few more comments:

The authors could give a little more introductory information about the physiological function of microvillous cells.

Figure 3A and 3C. Figure 3C shows the same data points as in 3A, right? I needed some time to understand this. The authors could state this more explicitly in the text of the manuscript.

Reviewer 2:

In general, authors have satisfactory replied to my comments and requests. However, as a final suggestion, I would be very cautious in outlining the role of olfaction in detecting denantonium (DEN) through the data obtained in methimazole treated and untreated animals. In KO mice (untreated), low concentrations of DEN did not induce attractive behavior as for WT mice, thus excluding, at a first glance, a role of the olfaction at least in these experimental conditions. If olfaction was involved in detecting DEN, I would expect KO mice (untreated) showing attractive behavior perhaps even more markedly than that observed in WT mice since a potential behavioral conflict (attraction/avoidance) caused by high and low concentration of DEN is absent in these mice. Perhaps, to have a better demonstration of the authors’ conclusions, it would have been useful to test if, in methimazole-treated WT-animals, DEN, at low concentration failed to induce attractive behavior.

Therefore, the conclusions, on the role of olfaction in the detection on DEN, have to be reconsidered because they are somehow contradictory and may generate confusion into a reader.
