# Supplementary figures and images for: The Nasal Solitary Chemosensory Cell Signaling Pathway Triggers Mouse Avoidance Behavior to Inhaled Nebulized Irritants
Source: eNeuro. 2023 Apr 10;10(4):ENEURO.0245-22.2023. doi: 10.1523/ENEURO.0245-22.2023 (PMC10101550; doi:10.1523/ENEURO.0245-22.2023)

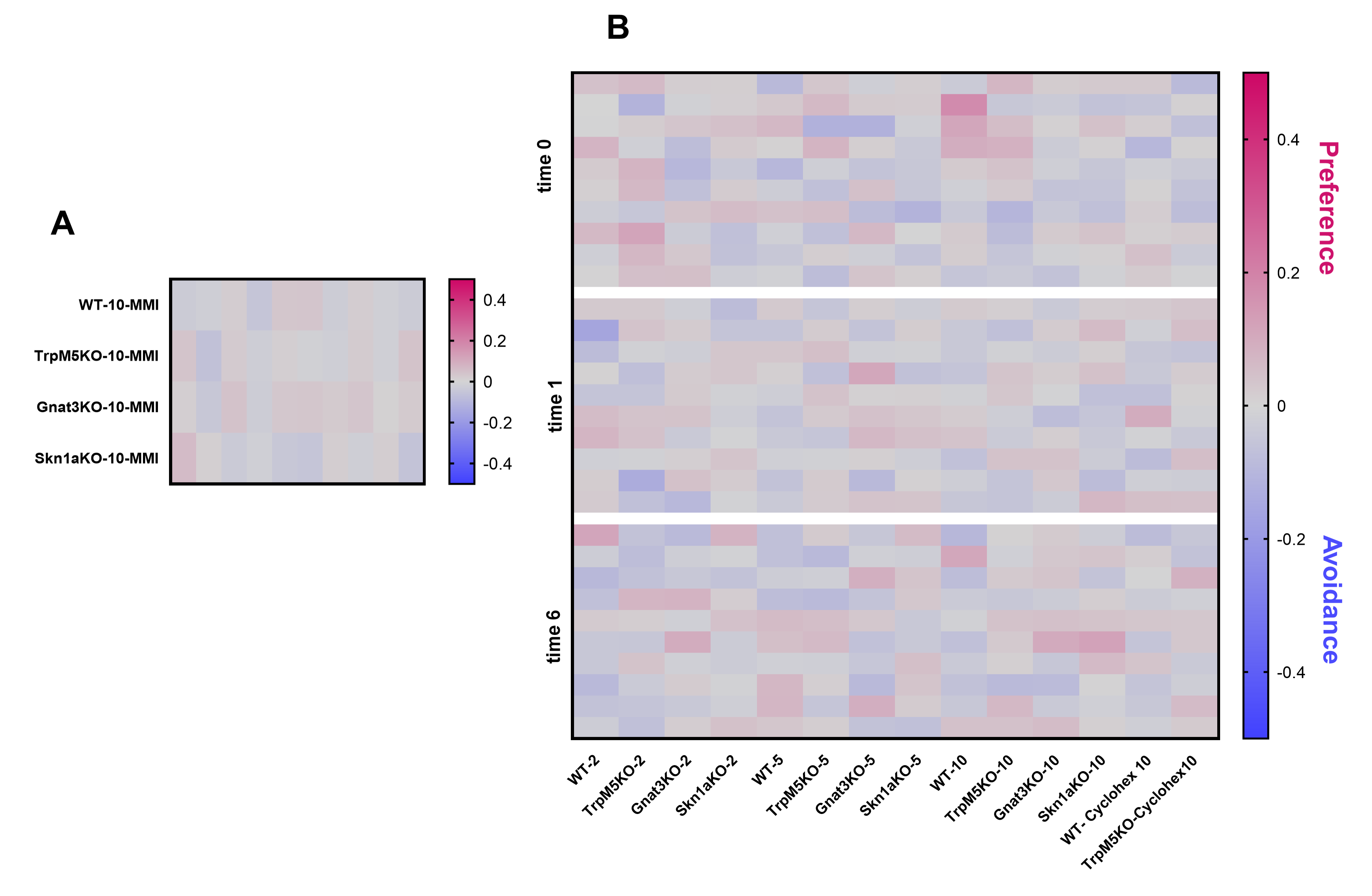

Supplement: Extended Data Figure 3-1 — Heat map representation of saline versus saline behavioral responses for all mice used in these experiments. A, Heat map of behavior scores for the 40 wild-type (WT) and knock-out (KO) mice exposed to saline in both chambers used for the olfactory epithelium ablation experiments with 10 mm denatonium (Den). B, Heat map for the 140 WT and KO mice exposed to saline in both chambers used for the dose-response (2, 5, and 10 mm Den) and repeated-exposure experiments (10 mm Den and 10 mm cycloheximide). Each rectangle represents the ratio of time the mouse spent in the left versus right chamber. Blue and red represent an increase in avoidance and preference, respectively, with color tones indicating intensity; gray is the baseline (behavior score = 0), representing indifference. Download Figure 3-1, TIF file. [file enu-eN-NWR-0245-22-s03.tif]

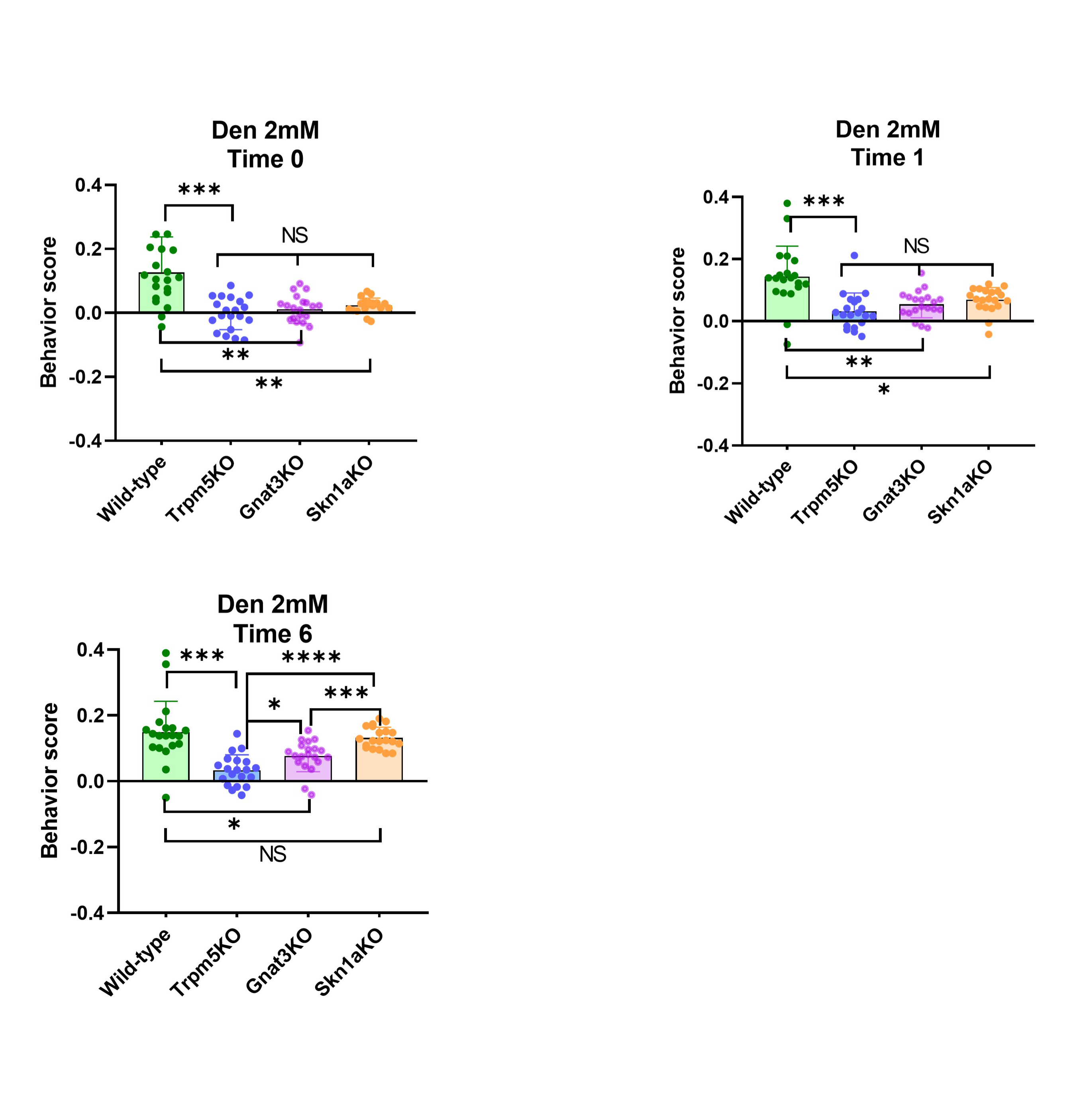

Supplement: Extended Data Figure 5-1 — Preference for 2 mm Den in both WT and SCC-KO mice. Both WT and Gnat3-KO, TrpM5-KO, and Skn1a-KO mice showed a consistent preference and/or improved behavior to 2 mm Den at all three time points (0, 1, and 6 h). n = 10 mice per group, 40 mice in total. Behavioral scores below 0 indicate avoidance of irritants and above 0 indicate attraction/preference. *0.01 < p ≤ 0.05, **0.001 < p ≤ 0.01, ***0.0001 < p ≤ 0.001, ****p < 0.0001, NS: no significance. Bars and symbols reflect mean ± SEM. Download Figure 5-1, TIF file. [file enu-eN-NWR-0245-22-s04.tif]

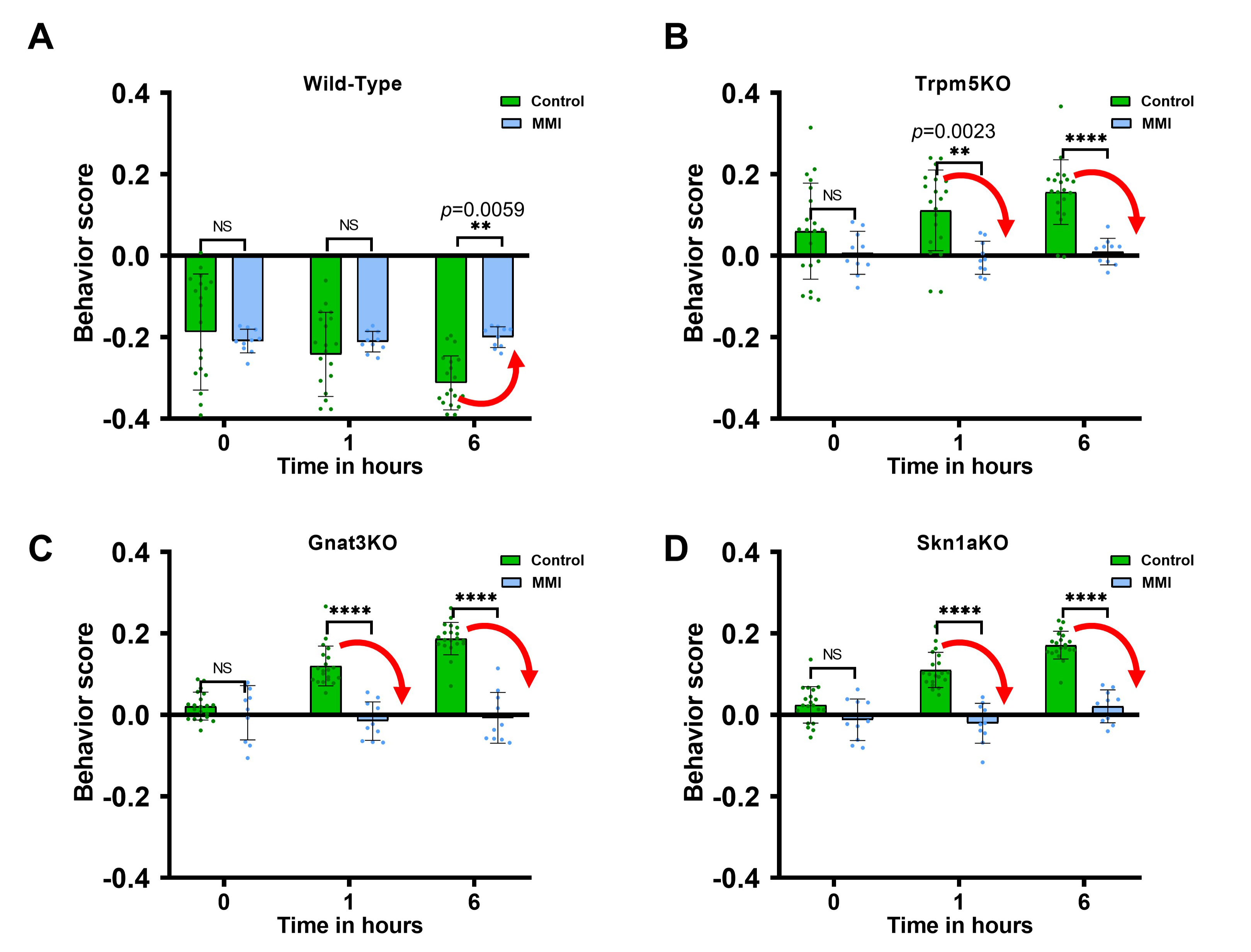

Supplement: Extended Data Figure 8-1 — Methimazole experiment data for mice injected with methimazole for each mouse strain and at each time point. After ablation of the sense of smell, both WT and KO mice lost the quicker avoidance and preference responses after methimazole injection (red arrows). n = 10 mice per group, 40 mice in total. Behavioral scores below 0 indicate avoidance of irritant and above 0 indicate attraction. **0.001 < p ≤ 0.01, ****p < 0.0001, NS: no significance. Bars and symbols reflect mean ± SEM. Download Figure 8-1, TIF file. [file enu-eN-NWR-0245-22-s05.tif]

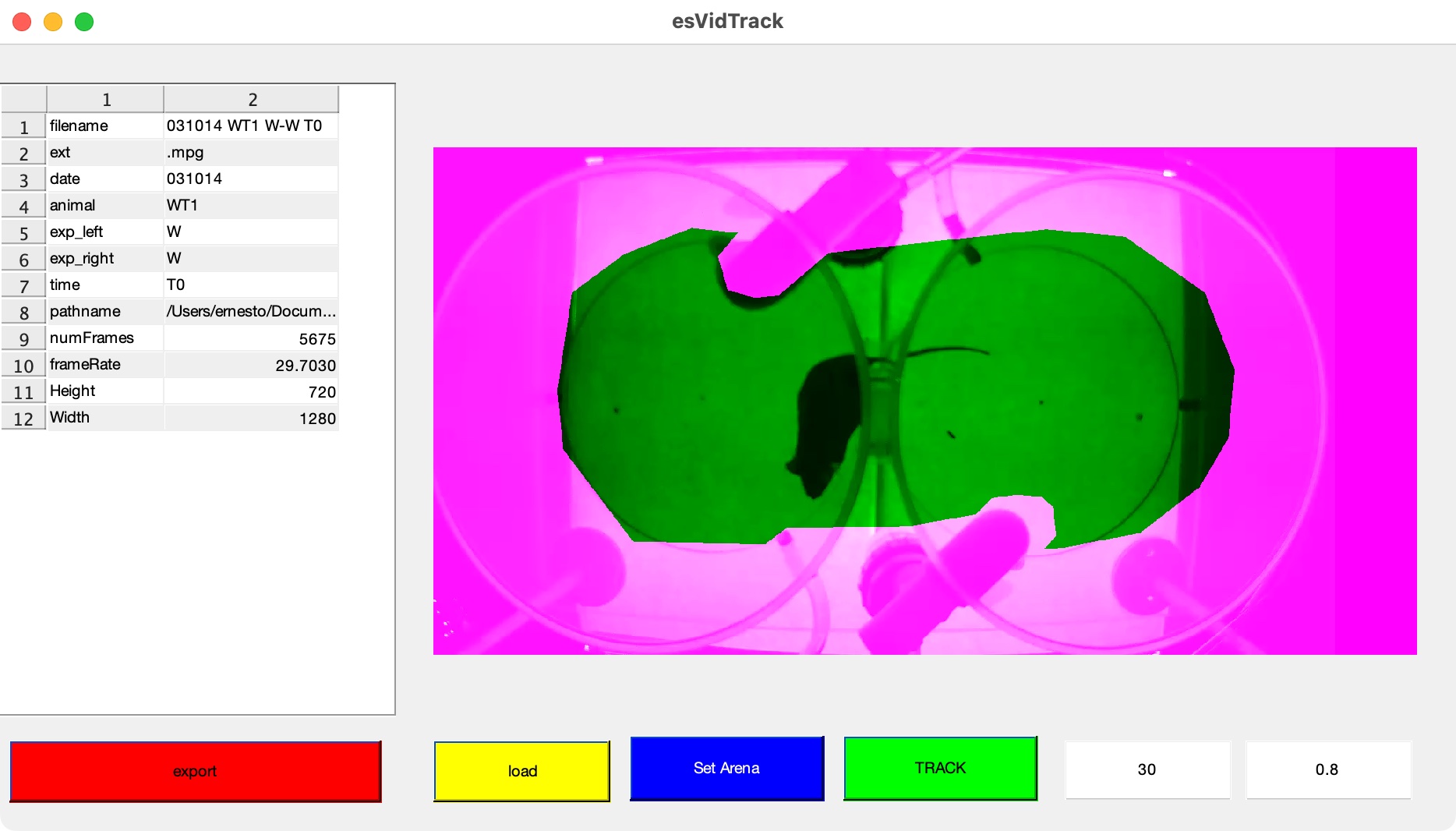

Supplement: Extended Data 1 — Zip file containing the MATLAB code, an example video used to track the behavior, and the example video showing typical behavior during a recording session. Download Extended Data 1, ZIP file. [file enu-eN-NWR-0245-22-s02.zip › T2R_mouse_vid_track-main/Example_Arena.jpg]
